# General deep learning framework for emissivity engineering

**DOI:** 10.1038/s41377-023-01341-w

**Published:** 2023-12-05

**Authors:** Shilv Yu, Peng Zhou, Wang Xi, Zihe Chen, Yuheng Deng, Xiaobing Luo, Wangnan Li, Junichiro Shiomi, Run Hu

**Affiliations:** 1 School of Energy and Power Engineering, Huazhong University of Science and Technology, Wuhan, 430074 China; 2 Hubei Key Laboratory of Low Dimensional Optoelectronic Materials and Devices, Hubei University of Arts and Science, Xiangyang, 441053 Hubei China; 3 Hubei Longzhong Laboratory, Wuhan University of Technology (Xiangyang Demonstration Zone), Xiangyang, 441000 Hubei China; 4 Department of Mechanical Engineering, The University of Tokyo, 7-3-1 Hongo, Bunkyo-ku, Tokyo 113-8654 Japan

**Keywords:** Metamaterials, Photonic devices

## Abstract

Wavelength-selective thermal emitters (WS-TEs) have been frequently designed to achieve desired target emissivity spectra, as a typical emissivity engineering, for broad applications such as thermal camouflage, radiative cooling, and gas sensing, etc. However, previous designs require prior knowledge of materials or structures for different applications and the designed WS-TEs usually vary from applications to applications in terms of materials and structures, thus lacking of a general design framework for emissivity engineering across different applications. Moreover, previous designs fail to tackle the simultaneous design of both materials and structures, as they either fix materials to design structures or fix structures to select suitable materials. Herein, we employ the deep Q-learning network algorithm, a reinforcement learning method based on deep learning framework, to design multilayer WS-TEs. To demonstrate the general validity, three WS-TEs are designed for various applications, including thermal camouflage, radiative cooling and gas sensing, which are then fabricated and measured. The merits of the deep Q-learning algorithm include that it can (1) offer a general design framework for WS-TEs beyond one-dimensional multilayer structures; (2) autonomously select suitable materials from a self-built material library and (3) autonomously optimize structural parameters for the target emissivity spectra. The present framework is demonstrated to be feasible and efficient in designing WS-TEs across different applications, and the design parameters are highly scalable in materials, structures, dimensions, and the target functions, offering a general framework for emissivity engineering and paving the way for efficient design of nonlinear optimization problems beyond thermal metamaterials.

## Abstract (web summary)

We proposed a general deep reinforcement learning framework to design materials and structures simultaneously for wavelength-selective thermal emitters with broad applications in emissivity engineering.

## Graphical abstract

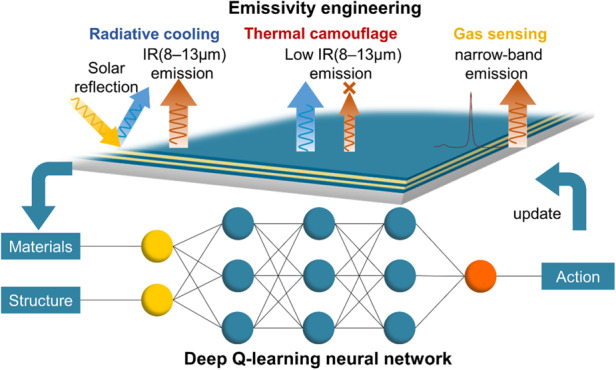

## Introduction

All objects in nature emit thermal radiation outwardly at anytime and anywhere in a broadband, non-selective, incoherent, diffusive, and reciprocal manner^1,2^. Thanks to the fast development of thermal metamaterials and metasurfaces in recent years, thermal radiation has been demonstrated to be engineered with comprehensive control of spectral, directional, and dynamic characteristics, enabling higher-efficiency radiative heat transfer than the thermal radiation of natural objects^3^. Among them, spectral emissivity engineering of thermal radiation enables more applications, such as energy harvesting^4^, thermal management^5,6^, radiative cooling^7,8^, thermal camouflage^9,10^, infrared (IR) sensing^11^, far-/near-field radiation control^12^, thermophotovoltaics^13^, thermography^14,15^, heat-assisted magnetic recording^16^, etc. The emissivity engineering aims to select materials and design nanostructures to achieve specific functionalities with a target emissivity spectrum. The common physics of selective emissivity comes from the excitation of different photon modes, which leads to the local enhancement or suppression of the internal electric field, thus allowing for control over the radiation emission at different wavelengths^17^. Wavelength-selective thermal emitters (WS-TEs), as the main output of emissivity engineering, can be achieved by multilayers^18^, photonic crystals, nano-grating^19^, nano antennas arrays^20^, multiple-quantum-well^21^, Fabry-Perot cavities^22^, hallow cavities^23^, etc. As one of the simplest structures of WS-TEs, one-dimensional multilayers are frequently employed which are composed of alternating layers of materials with different refractive indices, allowing or blocking the propagation of light of specific wavelength in it, and together with absorption of lossy medium, so as to achieve the regulation of emissivity^24^. The diversity of materials and the large parameter space of multilayer structures provide significant flexibility in tuning emissivity. Additionally, they are relatively easy to fabricate using thin film deposition at a low cost, which makes them promising for large-scale manufacturing. More importantly, the emissivity spectra of the multilayers can be efficiently simulated using the transfer matrix method (TMM), which is easily combined with various optimization algorithms. Therefore, multilayers are frequently designed as typical WS-TEs and are widely applied for extensive applications in thermal camouflage (TC)^25,26^, radiative cooling (RC)^27–29^, gas sensing (GS)^30–32^, etc.

**Fig. 1:**
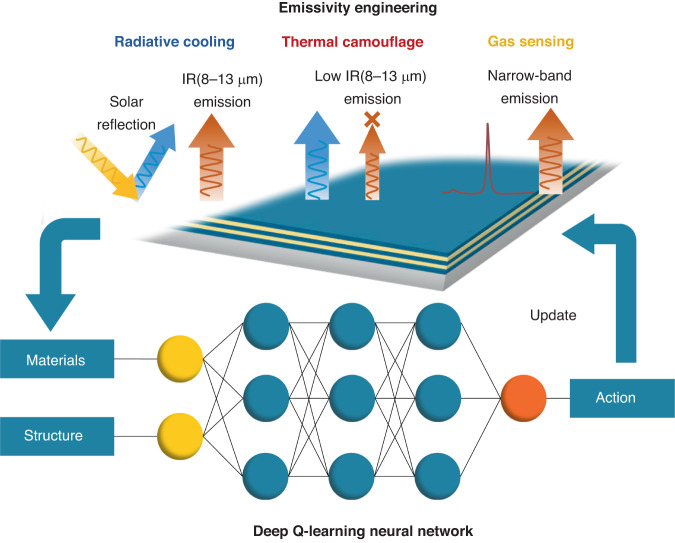
Emissivity engineering of multilayer WS-TEs designed and optimized by Deep Q-learning network (DQN) for radiative cooling, thermal camouflage, and gas sensing, respectively. The schematic for the emissivity requirement for different applications are included. The basic elements for the DQN network are also illustrated

In general, different applications require distinct emissivity spectra, as illustrated in Fig. 1. For instance, TC necessitates low emissivity within the long-wavelength IR range (8–13 μm) to prevent detection by most IR detectors when the background temperature is low, where the long-wavelength IR range is called as atmosphere window (AW) due to its high transmittance. Additionally, it is advantageous for the emissivity outside the AW to remain as high as possible to facilitate further radiative heat dissipation^33^. To achieve TC, Peng et al. designed a silver/germanium (Ag/Ge) multilayered structure, where impedance matching is utilized to manipulate the radiation characteristics^34^. Zhu et al. designed a Ge/ZnS multilayer on a silica aerogel substrate with efficient radiative cooling capability for TC in high ambient temperatures^35^. In contrast, RC aims to achieve passive cooling by radiating the heat directly to the outer space at ~3 K via the high emissivity within the AW. In addition, a high reflectivity in the solar band is necessarily required to reflect as much solar energy as possible to maximize the cooling power, ultimately achieving net energy outflow and reducing object temperature^36^. Raman et al. adopted needle optimization method to design a seven-layer HfO_2_/SiO_2_ emitter. The fabricated multilayer emitter achieved daytime RC under direct solar irradiance for the first time, which reflected 97% of solar irradiance and cooled to 4.9 °C below the ambient temperature^37^. Similarly, Ma et al. optimized seven-layer SiO_2_/Si_3_N_4_ emitter using an evolutionary algorithm, and the emitter was highly reflective towards solar radiation and had a broadband high emissivity within the AW^38^. Different from the broadband emissivity spectra for RC and TC, the emissivity spectrum for GS needs narrow-band peaks which match the absorption peaks of the detected gas. Sakurai et al.^39^ and Xi et al.^31^ both utilized machine-learning to design and optimize multilayered structures and achieved ultra-narrowband emission peaks at multiple wavelengths. In particular, Xi et al. obtained the whole database of multilayered WS-TEs for narrowband emissivity spectra in the wavelength range of 3 to 10 μm, and the highest *q*-factor reaches 508 far beyond the *q*-factor record in the literature.

As far as we are concerned from the literature, although many combinations of materials of multilayer WS-TEs have been proposed to regulate emissivity, both material selection and structural design still rely on physics-inspired methods and past design experience or guidelines, which are inefficient and difficult to achieve optimal structural design. To further improve the performance of multilayer WS-TEs, machine-learning optimization algorithms have shown unique advantages in structure optimization and designing problems^31,40^. However, designers still have to conduct extensive searches in existing work to determine suitable materials and initial structural parameters for their design goals before optimization. Consequently, researchers, following their prior knowledge of materials or structures for different applications, either fix materials to design structures^11,39,41^ or fix structures to optimize material arrangement^13,42^ to reduce the optimization space and improve the design efficiency. Hence, one open question comes that can we offer a general framework for designing WS-TEs for different applications without a prior knowledge of materials and structures? If so, we can just change the target emissivity spectra and the WS-TEs will be output directly with matching emissivity spectra to the target one.

Recently, deep learning has attracted increasing attention in various domains, such as natural language processing, computer vision, image processing, speech recognition and material structure optimization^43^. Through establishing the artificial neural network and data-driven method, deep learning obtains the mapping relationship between data pairs, that is, from emissivity spectra to design parameters of the emitters. However, challenges such as the one-to-many mapping problem, analysis from complex spectra to design parameters, along with the dataset acquisition, collectively render most neural network models inefficient for addressing the emitter design within an enormous optimization space that concurrently encompasses material selection and structural optimization simultaneously. Fortunately, deep reinforcement learning (DRL), which combines deep learning and reinforcement learning, promises to address the above challenges. It does not directly parse the mapping relationship between data pairs from the pre-collected dataset, but constantly interacts with the current environment to make decisions to update the state of the environment, and uses historical experience as the dataset to learn and optimize the deployment of decisions, so as to maximize the accumulated reward value^44^. Consequently, it has been proven to be capable of solving large-scale and complicated tasks, such as Go and Chess^45^. Wang et al. proposed a sequence generation network based on DRL for the design of optical multilayer films^46^. However, due to the design parameters being generated from the same network, their diversity is limited. In addition, other DRL based design frameworks still face serious challenges in terms of design efficiency^47^.

In this study, we propose a general design framework based on deep Q-learning network (DQN) for the design and optimization of WS-TEs in emissivity engineering without a prior knowledge of materials and structures. This framework demonstrates high accuracy and efficiency as well as flexibility and scalability in design parameters and applications. Three multilayer WS-TEs for three applications including TC, RC, and GS, are designed and optimized by the framework, which are then experimentally fabricated and measured, matching with the designed emissivity spectra. The selection of materials and the design of the structure are independently completed by DQN within the extensive optimization space. The designed multilayer WS-TEs all exhibit exceptional performance in these three applications, validating DQN as a general deep learning framework for emissivity engineering.

## Results

### Construction of DQN framework for WS-TEs design and optimization

**Fig. 2:**
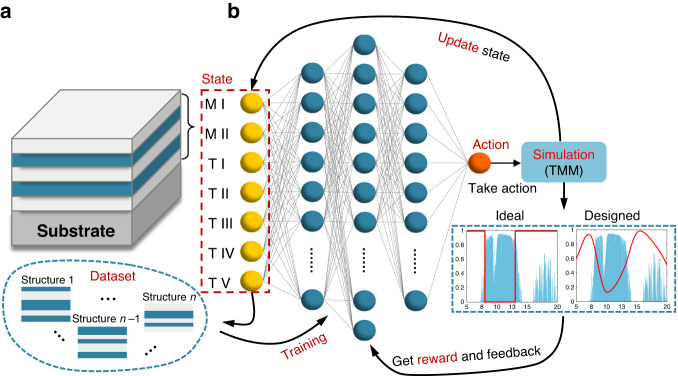
Schematic for the multilayer structure and DQN framework. **a** Five-layer multilayer structure composed of two alternating materials. **b** Schematic of the DQN framework. The state consists of two materials and five thicknesses of the multilayer, then the state parameters are fed into the DQN to generate an Action. Then take the action to update the state. TMM is adopted to simulate the new state, and reward is obtained to feed back to neural network (agent). The new state is fed into the DQN for the next iteration. Each pair of state, action and reward is recorded as dataset to train the neural network, so that it can take the action that increases accumulated reward and finally get the corresponding state with the maximum reward

The roadmap of optimization process of DQN is illustrated in Fig. 2. The whole optimization process can be described as an interactive process with the environment. The state of the environment, which consists of the material ID number and the thicknesses of each layer, represents the materials and structural parameters of the current multilayer. Here, we set up the multilayer WS-TEs as a 5-layer structure composed of alternating two materials. Considering that this specific structural configuration has been implemented for various applications in emissivity engineering^41,48^. Naturally, the setting of design parameters is flexible and can be adjusted according to design objectives, including the kinds of materials, layer count, and other structural parameters (For more details, see Supplementary Information Note 3). It is worth mentioning that, while increasing the number of layers and materials may meet more rigorous emissivity spectrum requirements, it also significantly expands the optimization space by several orders of magnitude, requiring greater computing power and longer design time. Consequently, according to the structural configuration set above, the state can be represented by a 1×7 vector containing material and structure information. The two materials are selected from the self-built material library, as shown in Table 1, which contains 8 commonly used materials for emissivity engineering. These candidate materials cover most optical properties. Their optical properties (refractive index) are referred to E. Palik’s and Querry’s books^49,50^ and other research work^51,52^ (See Supplementary Information S1). Regarding the substrate material, it needs to be selected according to specific design goals, we chose silver for RC, silica for TC, and tungsten for GS. Each layer thickness is varied within the range of 20–1000 nm with a uniform step size of 20 nm, which results in a total of 50 possible steps for each layer. Considering the 8 available materials, this structural configuration leads to 8 × 7 × 50^5^ = 1.75 × 10^10^ potential candidate structures. The demand of simultaneous material selection and structure optimization, together with the sheer volume of optimization space, renders manual design impractical and presents significant challenges to conventional machine learning methods.

**Table 1:** Material library of multilayer WS-TEs for TC, RC, and GS

Material ID	Material
1	Ge
2	ZnSe
3	Si
4	SiO_2_
5	TiO_2_
6	ZnS
7	Si_3_N_4_
8	MgF_2_

After the physical information of the multilayer structure is encoded into digital information, it is inputted into an artificial neural network. The network, called ‘agent’ in DQN, consists of an input layer, three fully connected layers and an output layer. The number of neurons in the three fully connected layers is 24, 48 and 24, respectively. These layers perform computations on the input data, extracting relevant features and learning patterns from the encoded structural information. The output layer of the agent is referred to as the “action” layer. It generates a single value and each value corresponds to a policy that can be applied to update the current state (structure). More details about the actions and their corresponding policies can be found in Table 2, which provides a mapping between the output values of the action layer and the structural modifications they represent. Then TMM is adopted to simulate the radiation characteristics of the new state (new structure), and obtain its emissivity according to Kirchhoff’s law. To evaluate the performance of the new state, a reward *R* is obtained from the emissivity spectra. The reward serves as feedback for the agent and plays a crucial role in determining the convergence direction of the DQN model. The specific definition of the reward will depend on the desired application or emissivity target, and further details regarding the reward for TC, RC, and GS will be provided later.

**Table 2:** Definitions of actions used in DQN

Action No.	Action Definition
0	Decrease the material ID of Material I by 1 (min 1)
1	Increase the material ID of Material I by 1 (max 8)
2	Decrease the material ID of Material II by 1 (min 1)
3	Increase the material ID of Material II by 1 (max 8)
4	Decrease the Thickness I by 20 (min 20)
5	Increase the Thickness I by 20 (max 1000)
6	Decrease the Thickness II by 20 (min 20)
7	Increase the Thickness II by 20 (max 1000)
8	Decrease the Thickness III by 20 (min 20)
9	Increase the Thickness III by 20 (max 1000)
10	Decrease the Thickness IV by 20 (min 20)
11	Increase the Thickness IV by 20 (max 1000)
12	Decrease the Thickness V by 20 (min 20)
13	Increase the Thickness V by 20 (max 1000)

In the DQN, a *Q*-function *Q* (*s*, *a*) is defined to represent the expected cumulative reward for taking the action *a* on state *s* and following the optimal policy thereafter. The agent is trained to approximate the *Q*-function to make the best choice of action to achieve higher reward by utilizing the replay buffer, which stores historical experiences (state, action, reward, and next state) during the interaction with the environment. To enhance the stability of training process, the dual network structure is utilized, where the main network (agent) is used to collect experiences and the target network, a copy of agent, is used to calculate the target *Q*-value based on Bellman equation as follows^53^:

1 $${y}_{t}={r}_{t}+\gamma \cdot Q({s}_{t+1},{a}^{\ast };{w}^{-})$$

where *r*_*t*_ is the reward, *γ* is the discount factor, ${a}^{\ast }=\text{arg}{\max }_{a}Q({s}_{t+1},a;w)$ represents the action selected by the main network that maximizes the *Q*-value. *w*^*-*^ and *w* are the weights of the target network and the main network, respectively. The update of the network parameters is achieved by the back-propagation algorithm to minimize the loss function, which is the mean squared error between the predicted *Q*-value and the target *Q*-value, as follow:

2 $$loss=MSE[{y}_{t}-Q(s,a;w)]$$

In addition, Epsilon Greedy Exploration (EGE) algorithm is employed to balance exploration and exploitation. Initially, DQN tends to generate action randomly, but gradually, as epsilon decreases, it relies on the Q-function for decision making. Finally, it is crucial to design an appropriate initialization method for the state to make DQN capable for multilayer optimization with high efficiency. Here we randomly initialize two materials of the state from the material library, with the thickness of each layer randomly generated with the range described above. Additionally, we introduce an iteration threshold, which servers to evaluate whether the iteration should continue. When the reward *R* of a state exceeds the iteration threshold, the state with the highest historical reward is chosen as the initial structure for the next iteration. For each iteration, DQN continues to accept the state, take the action, simulate the emissivity spectra, feedback and then accept the next state. Once the reward of a new state falls below the iteration threshold, the structure will be reinitialized for the next iteration. It is important to note that the ‘train from buffer’ mechanism results in the number of simulations or the number of calculated structures are not equal to the number of iterations. In simple terms, the design and optimization process of DQN can be likened to playing a game. The game will continue until the mission fails, at which point it needs to be initialized and restarted. An ingenious initialization method can help achieve higher scores efficiently.

In order to showcase the generality and effectiveness of the DQN algorithm, we design multilayer WS-TEs in the following for three applications in emissivity engineering, including TC, RC, and GS, respectively, under the same optimization framework and utilizing a common material library.

### Design and optimization of WS-TE for TC

As mentioned earlier, the reward function needs to be meticulously defined to ensure that the optimization progress in the desired direction. So firstly, for TC, since an ideal TC emitter requires low emissivity inside AW (8–13 μm) but high emissivity outside, we therefore define the reward *R* as the difference between the average emissivity inside and outside the AW, which can be calculated as:

3 $$R=\frac{{\int }_{5}^{8}\varepsilon (\lambda ){I}_{{\rm{BB}}}(\lambda ,T)d\lambda +{\int }_{13}^{20}\varepsilon (\lambda ){I}_{{\rm{BB}}}(\lambda ,T)d\lambda }{{\int }_{5}^{8}{I}_{{\rm{BB}}}(\lambda ,T)d\lambda +{\int }_{13}^{20}{I}_{{\rm{BB}}}(\lambda ,T)d\lambda }-\frac{{\int }_{8}^{13}\varepsilon (\lambda ){I}_{{\rm{BB}}}(\lambda ,T)d\lambda }{{\int }_{8}^{13}{I}_{{\rm{BB}}}(\lambda ,T)d\lambda }$$

where ${I}_{{\rm{BB}}}=h{c}^{2}/{\lambda }^{5}\cdot {[\exp (hc/\lambda {k}_{{\rm{B}}}T)-1]}^{-1}$ is the spectral radiance of a blackbody at wavelength *λ* and temperature *T*. *h* and *k*_B_ are the Planck’s constant and Boltzmann constant, respectively and *c* is the speed of light. $\varepsilon (\lambda )$ is the emissivity spectrum of the designed TC emitter. The temperature here is set to 350 K, which is slightly higher than the average surface temperature of armored vehicles in the military. The reward *R* yields a value between 0 and 1 based on Eq. (3). By pre-trial, the iteration threshold is set as 0.2. In addition, the rewards *R* less than 0.2 are mandatorily modified to −0.2, which signals to the agent that the states corresponding to the negative rewards do not meet the design requirements. The initialization method may introduce randomness to the optimization results or lead the optimization to a local optimal solution. To mitigate the above impact, the optimization process is run 5 times to obtain the optimal TC emitter structure. Each run consists of 1000 iterations, which is sufficient to reduce epsilon in the Epsilon Greedy algorithm to its minimum value. This ensures that the agent dominates the selection of actions. Once the optimization is completed, the optimal structure is experimentally fabricated using magnetron sputtering to demonstrate the feasibility of the structural optimization.

**Fig. 3:**
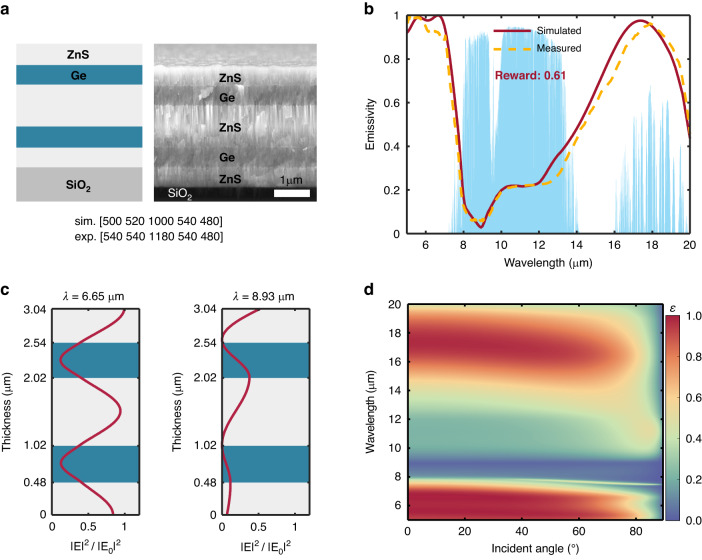
Results of TC emitter designed by DQN. **a** Schematic and SEM image of the optimal TC structure. ZnS and Ge are chosen as the materials and the layer thicknesses of simulation and experiment are presented. **b** Emissivity spectrum of the optimal TC emitter. **c** Normalized electric field intensity for optimal TC emitter at various wavelengths (*λ* = 6.65, 8.93 μm). **d** Emissivity as the function of incident angle and wavelength

The schematic of resulting optimal structure and corresponding scanning electron microscopy (SEM) image of fabricated multilayer are shown in Fig. 3a. It can be seen that DQN finally choose ZnS and Ge as the materials for the TC emitter. The thicknesses of each layer, including the values designed and those obtained from the SEM image of the fabricated sample, are presented in Fig. 3a. It is evident that the layer thicknesses in the optimal TC emitter are irregular and aperiodic, which is difficult to design accurately for manual optimization. However, due to the manufacturing precision, there are certain deviations between the thicknesses of fabricated sample and the designed values, resulting in the discrepancy of their corresponding emissivity spectra as depicted in Fig. 3b. In addition, the differences between the optical properties of the sputtered materials used for fabrication and the input parameters used in the numerical simulation also make a certain impact. Nevertheless, both the designed and fabricated structures exhibit low emissivity within the AW and high emissivity outside the window. The calculated average normal emissivity in AW of simulation is 0.18, while 0.79 is obtained outside the AM, resulting in the reward value of 0.61. The excellent camouflage effect is attributed to low thermal emission in the AW (IR camera detected band) and high emission outside AW for further radiative cooling. For further verification, the normalized electric field intensities of the optimal structure at 6.65 μm and 8.93 μm are plotted in Fig. 3c. The intensity of the electric field at 8.93 μm is degraded heavily, which means a forbidden band is formed in AW resulting in low absorption (and therefore low emissivity) in this band. While the intensity outside AW remains relatively unchanged, resulting in high emissivity for the structure with the lossy SiO_2_ substrate. The emissivity of the optimal structure as a function of incident angle and wavelength is shown in Fig. 3d, indicating the angular independence of the excellent performance.

**Fig. 4:**
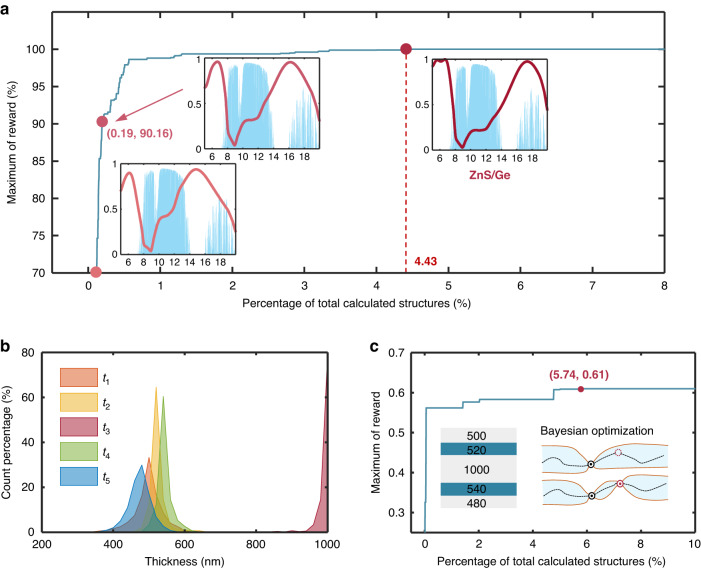
Optimization process for TC emitter by DQN. **a** Maximum of reward *R* as a function of the percentage of calculated structures for TC by DQN. **b** The parametric distribution curves of each layer thickness. **c** Maximum of reward *R* as a function of the percentage of calculated structures for TC by BO

In order to demonstrate the efficiency of the optimization under the framework of DQN algorithm, we quantitatively analyze the reward *R* as a function of the percentage of the number of calculated structures. As shown in Fig. 4a, DQN only calculated less than 0.2% of all the calculated structures to obtain 70% and 90% of the maximum reward and calculated only 4.428% of the structures to find the optimal structure for TC. It can be obviously seen that, with the progress of optimization iterations, the emissivity within the AW decreases continuously, while the emissivity outside the window gradually increases, aiming to achieve a better camouflage effect. In addition, the material combinations of structures achieving 70% and 90% of the maximum reward are the same as the optimal structure, as shown in Fig. S2, which indicates that DQN is capable of selecting appropriate materials at a rapid pace and then performs subsequent structural optimization. The parametric distribution curves of each layer thickness are presented in Fig. 4b, which indicate that the optimal layer thicknesses are derived from the peak of the curves. To further confirm the optimality of the structure obtained, we perform Bayesian optimization (BO) on the multilayer WS-TEs for TC under the fixed material combination, namely ZnS and Ge. Figure 4c illustrates the reward histories, showing that the maximum reward and corresponding structure by BO are consistent with those obtained using DQN. However, the proposed framework based on DQN still demonstrates higher efficiency while optimizing both materials and structure. Further details on BO for TC emitter are available in Supplementary Information Note 1.

### Design and optimization of WS-TE for RC

For designing a RC emitter, the objective is to maximize the emissivity within the AW, while minimizing it in the solar band so as to achieve maximum net energy power outflow. The net energy power, also called cooling power, can be denoted by

4 $${P}_{{\rm{cooling}}}(T)={P}_{{\rm{rad}}}(T)-{P}_{{\rm{atm}}}({T}_{{\rm{amb}}})-{P}_{{\rm{sun}}}(\theta )-{P}_{{\rm{cond}}+{\rm{conv}}}$$

where ${P}_{{\rm{rad}}}$ is the output power from the RC emitter, ${P}_{{\rm{atm}}}$ is the input power from the atmosphere radiation, ${P}_{{\rm{sun}}}$ is the input power from the sun and ${P}_{{\rm{cond}}+{\rm{conv}}}$ describes the heat exchange between the RC emitter and the environment by conduction and convection. *T* and *T*_amb_ are the temperature of RC emitter and ambient, respectively. $\theta$ is the angle of solar radiation. A more detailed calculation method of each power is provided in the Supplementary Information Note 2. In the following calculation, the conjugate heat transfer coefficient in ${P}_{{\rm{cond}}+{\rm{conv}}}$ is set as ${h}_{{\rm{c}}}=5W\cdot {m}^{-2}\cdot {K}^{-1}$ and the ambient temperature is kept at ${T}_{amb}=25\,^\circ {\rm{C}}$ to simulate a breeze situation. Obviously, the greater the cooling power, the better the performance of the designed RC emitter. However, it seems not intuitive to use cooling power as reward, and it is difficult to set a suitable iteration threshold. Therefore, the reward *R* is set as the difference between the steady-state temperature (*T*_steady_) of the RC emitter and the ambient temperature, namely the temperature drop below the *T*_amb_. If the ${P}_{{\rm{cooling}}}$ is positive at the initial temperature ${T}_{{\rm{init}}}$ (${T}_{{\rm{init}}}$=${T}_{{\rm{amb}}}$), the RC emitter starts to be cooled down. As the temperature of cooler decreases, the cooling power ${P}_{cool}$ also reduces until ${P}_{{\rm{cooling}}}({T}_{{\rm{steady}}})=0$. At that time, the RC emitter reaches an equilibrium state and the *T*_steady_ can be obtained from the Eq. (4)^27^. Previous studies have shown that the temperature difference ($\varDelta T={T}_{{\rm{amb}}}-{T}_{{\rm{steady}}}$) can reach 8 °C or even higher^8,36^, so the iteration threshold is set as 5 °C. Similar to the previous design for TC, the rewards *R* less than 5 will be mandatorily modified to −5. The optimization is also implemented for 5 times with 1000 iterations each.

**Fig. 5:**
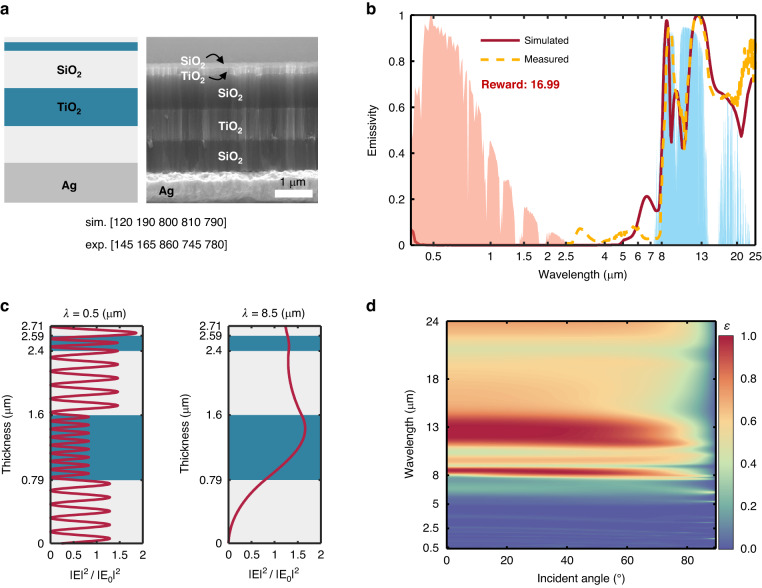
Results of RC emitter designed by DQN. **a** Schematic and the SEM image of the optimal RC structure. TiO_2_ and SiO_2_ are chosen as the materials and the layer thicknesses of simulation and experiment are presented. **b** Emissivity spectrum of the RC emitter. **c** Normalized electric field intensity for optimal emitter at various wavelengths (*λ* = 0.5, 8.5 μm). **d** Emissivity as the function of incident angle and wavelength

The design and optimization results of the RC emitter are presented in Fig. 5a. SiO_2_ and TiO_2_ are finally chosen as the materials for the optimal RC structure. The layer thicknesses of the optimal RC emitter also exhibit irregular and aperiodic. The emissivity spectra of the designed and the fabricated structures are shown in Fig. 5b. It can be seen that the designed RC emitter exhibits near zero emissivity in solar spectrum band, allowing it to reflect the incident solar radiation energy. In contrast, a high emissivity is obtained within the AW, enabling it radiates heat efficiently to outer space. Due to the differences between the thickness of the fabricated sample and the designed values, their emissivity spectra are not completely consistent. The reward *R* of the optimal RC emitter is 16.99, which means it can maintain 16.99 °C below the ambient temperature at thermal equilibrium in theory. The cooling power at the initial temperature is 132.40 W/m^2^. The equilibrium temperature difference and cooling power both exhibit the excellent performance of the designed RC emitter. The normalized electric field intensities of the optimal structure in the visible wavelength band and AW are illustrated in Fig. 5c, indicating the strong reflection of the Ag substrate and the high emissivity resulting from the electric field enhancement, respectively. Furthermore, the angular independence of emissivity spectra can also be observed within an angle of less than 80°, as shown in Fig. 5d.

**Fig. 6:**
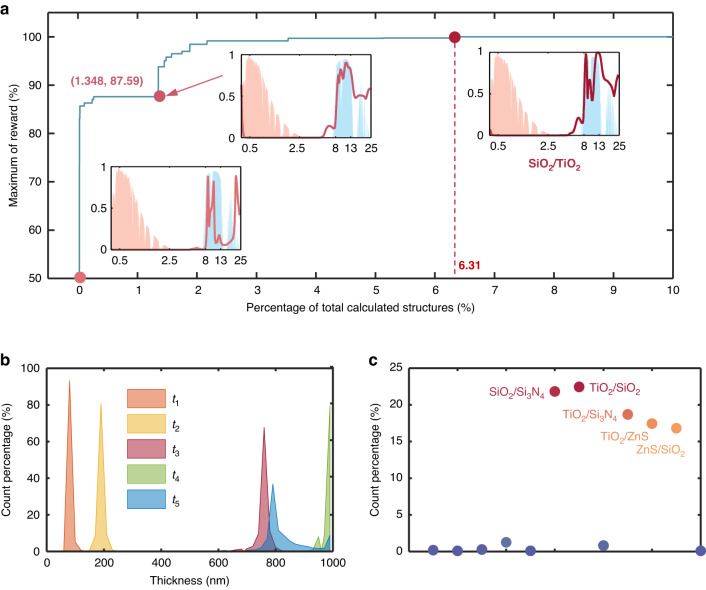
Optimization process for RC emitter by DQN. **a** Maximum of reward *R* as a function of the percentage of calculated structures for RC. **b** Parametric distribution curves of each layer thickness. **c** Distribution of material combinations except SiO_2_/TiO_2_

The optimization process is quantitatively shown in Fig. 6a. In the early stage of optimization, the reward *R* increases sharply, which means that DQN can quickly identify suitable materials for the RC emitter and perform optimization under this material combination until the optimization process tends to be smooth (as shown in Fig. S3). The material combination of the structure yielding 50% of maximum reward is Si/SiO_2_, which indicates that DQN replaces Si with TiO_2_ to achieve better cooling performance, as shown in Fig. S3a. During the smooth optimization period, the thickness of each layer is continuously optimized to further enhance the radiative cooling performance. When calculating less than 2% of the candidate structures, the RC emitter could reach a temperature drop of 14.94 °C below the ambient temperature in a steady state. After 1000 iterations, only 6.31% of structures need to be calculated to find the structure for the RC emitter with the maximum reward. To further exhibit the details of the optimization, the parametric distribution curves of each layer thickness are shown in Fig. 6b. In addition, except for the material combination of the optimal RC emitter, other material combinations are shown in Fig. 6c. It can be seen that SiO_2_ and Si_3_N_4_ also exhibit potential as the materials of RC emitter, in addition to TiO_2_ and SiO_2_. The occurrence of less frequent material combinations can be explained by the random initialization of the DQN and the random selection of the EGE algorithm used in DQN.

### Design and optimization of WS-TE for GS

In the final part of this work, we adopt DQN to tackle a more rigorous task, that is, to achieve peak emissivity at a fixed wavelength for GS. More specifically, the target is to obtain a narrow-band emission peak with a high emissivity at the wavelength of absorption peak of the detected gas, while the emissivity at other wavelengths is zero to eliminate the impact of absorption by other gases. Here, we take carbon dioxide (CO_2_) as the target gas, which has an absorption peak at 4.26 μm. The reward is defined as the product of the emissivity at 4.26 μm and the *q*-factor of the narrow peak, as follows:

5 $$R={\varepsilon }_{{\rm{t}}}\times q$$

where *q* is used to ensure that a narrow-band emission peak can be generated and ${\varepsilon }_{{\rm{t}}}$ is to ensure a high emissivity at target wavelength, 4.26 μm. Maximize the product of the two terms to optimize the resulting GS emitter with a narrow-band emission peak that matches the carbon dioxide absorption peak. By pre-train, the iteration threshold is set to 2. The optimization was run 5 times with 1000 iterations each to obtain the optimal structure while eliminating randomness.

**Fig. 7:**
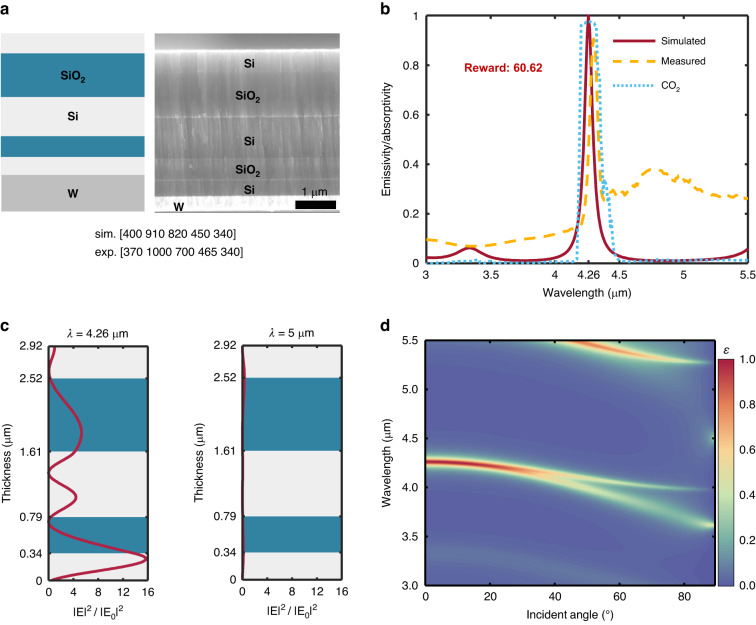
Results of GS emitter designed by DQN. **a** Schematic and the SEM image of the optimal GS structure. Si and SiO_2_ are chosen as the materials and the layer thicknesses of simulation and experiment are presented. **b** Emissivity spectrum of the GS emitter. **c** Normalized electric field intensity for optimal emitter at various wavelengths (*λ* = 4.26, 5 μm). **d** Emissivity as the function of incident angle and wavelength

As shown in Fig. 7a, the Si and SiO_2_ are chosen as the materials of GS emitter by DQN. The emissivity spectra of the optimized structure are shown in Fig. 7b. The simulation result shows that a sharp and high emissivity peak can be realized with the optimized structures at 4.26 μm, and the emissivity outside the narrow-band is close to zero. The corresponding emissivity of the peak is 0.9996, and the reward *R* of the structure is 60.62. The result shows that the designed WS-TE is sufficient to be an excellent CO_2_ sensor. Due to the thickness deviation of the fabricated sample, the measured wavelength of the emissivity peak deviates from the target wavelength but still within the CO_2_ absorption peak. The emission peak is located at 4.3 μm and the peak value is 0.905. Regrettably, the fabricated sample presents a certain low emission outside the absorption peak, which can be attributed to the discrepancy in properties between the sputtered and simulated materials. Figure 7c displays the normalized electric field intensities of the optimal structure at 4.26 μm and 5 μm. Due to the excitation of the localized Tamm plasmon state, the electric field intensity is significantly enhanced in a region 0.3 μm from the top of the substrate, resulting in peak emissivity at 4.26 μm. However, there is no notable enhancement of the intensity of electric field at 5 μm, resulting in near-zero emissivity at this wavelength. The incident angle related emissivity spectra are displayed in Fig. 7d. It can be seen that the angular independence only occurs within 30°, but it does not have any effect on gas sensing since the emitter typically faces the detected gas in the normal direction.

**Fig. 8:**
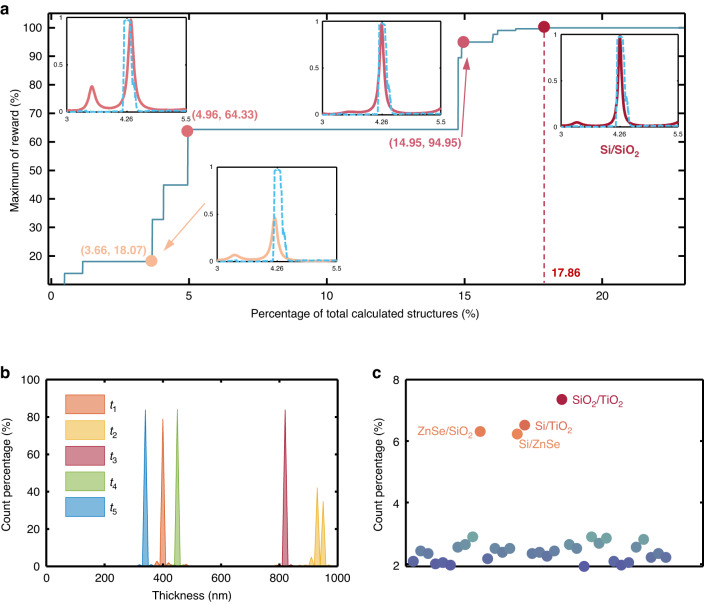
Optimization process for GS emitter by DQN. **a** Maximum of reward *R* as a function of the percentage of calculated structures for GS. **b** Parametric distribution curves of each layer thickness. **c** Distribution of material combinations except Si/SiO_2_

The optimization process of the GS emitter is presented in Fig. 8a. In the early stage of optimization, the emitter has only a small emissivity peak within the research band and the wavelength of emissivity peak deviates from 4.26 μm. As the iterations progress, suitable material combinations and optimized structure parameters lead to an improved and more obvious emissivity peak that gradually shifts towards the target wavelength of 4.26 μm. Eventually, a near perfect emissivity peak is achieved at 4.26 μm with a *q*-factor of 60.64. Further insights into the structure evolution during the optimization process can be obtained from Fig. S4. The distribution of each layer thickness as well as the material combinations are shown in Figs. 8b, c, respectively. Note the formation of peaks in the layer thickness distribution and the diversity of material combinations, indicating that appropriate material combinations are more important for achieving finer emissivity spectra. DQN successfully recognized this feature and efficiently implemented the design of the emitter with the help of the defined initialization method. Consequently, the combination of Si and SiO_2_ is undoubtedly the most suitable choice for achieving the target emissivity spectrum for CO_2_ sensing.

## Discussion

In summary, we present a general deep learning framework, i.e., DQN, for emissivity engineering of WS-TE design across applications. To demonstrate the generality, three WS-TEs are designed for typical applications, namely TC, RC and GS, which can autonomously select suitable materials from the same self-built material library for different design target functions and optimize to output the best structural parameters from a huge optimization space efficiently. The three design tasks are based on the same structural framework, so they can share the same material library, and can be easily extended from application to application by setting the corresponding reward function. The merits of the deep Q-learning algorithm include that it can (1) offer a general design framework for WS-TEs beyond one-dimensional multilayer structures, such as two-dimensional periodic array and complicated structures; (2) autonomously select suitable materials from a self-built material library without presetting the initial materials, and (3) autonomously optimize structural parameters for the target emissivity spectra efficiently across different applications. Additionally, the input parameters of the DQN framework are highly flexible in materials, structures, dimensions, and the target functions, paving a general solution to other nonlinear optimization problems beyond emissivity engineering.

## Materials and methods

### Simulation

The reflection and transmission of the multilayer WS-TEs were calculated using transfer matrix method based on the Fresnel equations. The emissivity was obtained from the corresponding reflection and transmission according to the law of conservation of energy. The code of DQN was written using the Keras package in TensorFlow and implemented in Python.

### Sample fabrication and preparation

The designed multilayer WS-TE samples were all deposited by a magnetron sputter (Kurt J. LesKer-VD75). The deposition rates of SiO_2_, TiO_2_, Ag, Si, W, Ge and ZnS are 2, 3, 6, 9, 3, 5, and 11 nm/min, respectively.

### Optical characterization

The infrared emissivity of the multilayer WS-TE samples was measured using a Fourier transform infrared spectrometer (Nicolet iN10, Thermo Scientific).

### Supplementary information

Final Supplementary Materials for optical properties and optimization details

## Data Availability

The data that support this research’s findings are available and can be provided based on the request to the corresponding authors.
