# Supplementary material for: General deep learning framework for emissivity engineering
Source: Light Sci Appl. 2023 Dec 5;12:291. doi: 10.1038/s41377-023-01341-w (PMC10697983; doi:10.1038/s41377-023-01341-w)
Supplement: Supplementary file 1 — Final Supplementary Materials for optical properties and optimization details [file 41377_2023_1341_MOESM1_ESM.docx]

Supplementary Information for

**General deep learning framework for emissivity engineering**

*Shilv Yu1, Peng Zhou2, 3, Wang Xi1, Zihe Chen1, Yuheng Deng2, 3, Xiaobing Luo1, Wangnan Li2, 3, *, Junichiro Shiomi4, *, Run Hu1, **

1School of Energy and Power Engineering, Huazhong University of Science and Technology, Wuhan 430074, China

2Hubei Key Laboratory of Low Dimensional Optoelectronic Materials and Devices, Hubei University of Arts and Science, Xiangyang 441053, Hubei, China

3Hubei Longzhong Laboratory, Wuhan University of Technology (Xiangyang Demonstration Zone), Xiangyang 441000, Hubei, China

4Department of Mechanical Engineering, The University of Tokyo, 7-3-1 Hongo, Bunkyo-ku,Tokyo 113-8654, Japan

*E-mail: [liwangnan@hbuas.edu.cn](mailto:liwangnan@hbuas.edu.cn); [shiomi@photon.t.u-tokyo.ac.jp](mailto:shiomi@photon.t.u-tokyo.ac.jp); [hurun@hust.edu.cn](mailto:hurun@hust.edu.cn)

**Content**

The optical properties of the materials in self-built material library (Fig. S1).

The detail of Bayesian Optimization for thermal camouflage emitter designing (Note 1).

The detail calculation of cooling power and sub-ambient temperature of radiative cooling emitter (Note 2).

The material and structure parameters of structures in the optimization process in Fig. 4, Fig. 6 and Fig. 8 (Fig. S2–S4).

Scalability of WS-TEs’ design parameters (Note 3 and Fig. S5–S7).

**The optical properties (refractive index and extinction coefficient) of the candidate materials in the self-built material library.**

**Fig. S1 |** **a** Refractive index (n) and **b** extinction coefficient (k) of the materials in self-built material library for WS-TEs in emissivity engineering: Ge 1, Si, SiO2 1, TiO2 2, ZnS 3, Si3N4 4, MgF2 4 and ZnSe 3.

These materials are commonly used in emissivity engineering, especially for the applications, such as thermal camouflage, radiative cooling, gas sensing, thermophotovoltaic, etc. Meanwhile, they cover most optical properties, e.g. high refractive index (Ge, Si), low refractive index (SiO2, MgF2), infrared (8–13 μm) absorbing (SiO2, TiO2, Si3N4) and infrared transparent (ZnSe, ZnS). Consequently, these candidate materials are sufficient and their combination enables flexible modulation of emissivity to meet spectral requirements under different applications.

**Note 1: Bayesian Optimization for thermal camouflage emitter**

The BO was performed with the help of the open-source Python library PHYSBO 5. The BO can select inputs that will yield better outputs from a list of candidate inputs with the help of training a Gaussian process. So, we need to create a list of all candidates before performing BO. If we perform BO under the same optimization space as described in the main text, the computer memory usage required to create candidate list and optimize hyperparameters will be extremely large, exceeding 500GB. To make the BO executable and for the purpose of verifying the optimal structure designed by DQN, we fixed the materials as ZnS and Ge and reduced each layer thickness range to 400 – 1000 nm. The number of iterations was set as 32588 to ensure that the total number of calculated structures in designing the TC emitter is the same as that of DQN, of which 500 are random searches as training data. The random feature map and Thompson sampling are utilized to avoid the computationally expensive training process. Although there is a significant difference between the set number of iterations (32, 588) and the total number of candidate structures (315 = 28,629,151), it was found during the optimization process that the optimal structure remained unchanged for a considerable period of time. Therefore, we can consider that the optimization has converged (Figure 4c).

**Note 2:** **Cooling power and steady-temperature calculation of radiative cooling**

Considering the WS-TEs of RC has an area *A* and a temperature *T*, facing the sky with the normal direction towards the zenith. In addition, the complex environment of the emitter is simplified, only considering the standard sun and breeze environment 6. With such setup, its cooling power *P*cooling can be described as:

$P_{\text{cooling}}(T)=P_{\text{rad}}(T)-P_{\text{atm}}(T_{\text{amb}})-P_{\text{solar}}(\theta)-P_{\text{cond+conv}}(T)$ (1)

where *P*rad is the power emitted from the emitter. *P*atm is the is the input power from the atmosphere absorbed by the emitter. *P*solar is the incident solar power, and the power of the non-radiative heat transfer due to the conductive and convective is described by the *P*cond+conv. *T* and *T*amb are the temperature of the emitter and the ambient air, respectively. *θ* is the angle of solar radiation. *P*rad is given by:

$P_{\text{rad}}(T)=A\int d\Omega\int_{0}^{\infty} I_{\text{BB}}(T,\lambda)\varepsilon(\lambda,\theta)\cos\theta d\lambda$ (2)

where *ε* (*λ*, *θ*) is the emissivity of the emitter at wavelength *λ* and angle *θ*. is the solid angle integration over a hemisphere and *I*BB (*T*, *λ*) is the spectral radiance of a blackbody at temperature *T* and wavelength *λ*, which is given by the Planck’s law:

$I_{\text{BB}}(\lambda,T)=\frac{2hc^{2}}{\lambda^{5}}\frac{1}{\exp(hc/\lambda k_{\text{B}}T)-1}$ (3)

where *h* is the Planck’s constant, *k*B is the Boltzmann constant and *c* is the speed of the light. The input power from the atmosphere radiation in Eq. (1) is given by:

$P_{\text{atm}}(T_{\text{amb}})=A\int d\Omega\int_{0}^{\infty} I_{\text{BB}}(T_{\text{amb},}\lambda)\alpha(\lambda,\theta)\varepsilon_{\text{atm}}(\lambda,\theta)\cos\theta d\lambda$ (4)

where *α* (*λ*, *θ*) is the absorption of the emitter at wavelength *λ* and angle *θ*. *ε*atm (*λ*, *θ*) is the emissivity of the atmosphere, which can be calculated as: $\varepsilon_{\text{atm}}(\lambda,\theta)=1-\tau(\lambda)^{1/\cos\theta}$, here *τ* (*λ*) is the transmittance of the atmosphere in the zenith direction. The input solar power is given by:

$P_{\text{solar}}(\theta)=A\cdot G\int_{0}^{\infty} \varepsilon(\lambda,\theta)I_{\text{AM}1.5}(\lambda)/\int_{0}^{\infty} I_{\text{AM}1.5}(\lambda)d\lambda$ (5)

where *I*AM1.5 (*λ*) is the standard AM 1.5 spectrum of solar radiation, and *G* is the total solar irradiance at 1 KW∙m­-2. The *P*cond+conv is given by:

$P_{\text{cond+conv}}=A\cdot h_{\text{c}}(T_{\text{amb}}-T)$ (6)

where *h*c is a non-radiative heat transfer coefficient that combines the effective conductive and convective heat exchange.

By integrating the above equations separately, we can obtain the cooling power *P*cooling of the emitter at different temperature *T*. When the emitter reaches a thermal equilibrium state, the *P*cooling is zero, and the corresponding steady-temperature *T*steady can be obtained.

**Structures in the optimization process**

**Fig. S2 | The structures obtained during the optimization process for TC**. **a** The structure with the 70% of the maximum reward. **b** The structure with the 90.16% of the maximum reward. Both of them are composed of ZnS and Ge, and the layer thicknesses are optimized by DQN to achieve better performance.

**Fig. S3 | The structures obtained during the optimization process for RC**. **a** The structure with the 50% of the maximum reward, which is composed of Si and SiO2. **b** The structure with the 87.59% of the maximum reward, consisting of three layers of TiO2 and two layers of SiO2, which is different from the optimal structure (Fig. 5) of three layers of SiO2 and two layers of TiO2. It is intuitive to see that the material and the structure are simultaneously optimized by DQN to achieve the optimal structural design.

**Fig. S4 | The structures obtained during the optimization process for GS**. **a** The structure with the 18.07% of the maximum reward, which is composed of TiO2 and Ge. **b** The structure with the 64.33% of the maximum reward, which is composed of Ge and ZnS. **c** The structure with the 94.95% of the maximum reward, which is composed of Si and SiO2, the same material combination as the optimal structure (Fig. 7). It is also intuitive to see that the material and the structure are simultaneously optimized.

**Note 3: Scalability of WS-TEs’ design parameters**

**The design of the multilayer structure with three or four materials as a basis**

In order to demonstrate the scalability of the proposed framework in terms of design paraments, we design three-, four-, six- and eight-layer emitters based on three or four materials to achieve thermal camouflage. The materials are chosen from the material library described in the main text. The thickness of each layer ranges from 200 to 800 nm. The optimization of each structural configuration is performed twice to reduce the effect of randomness. The results are presented as follows.

The three-layer structure is composed of Ge, TiO2 and MgF2, and the thickness of each layer is also shown in Fig. S5a. Its corresponding emissivity spectrum is also shown (Fig. S5a middle), and the reward calculated according to Eq. (3) in the main text is 0.4535. Although it shows a certain effect of thermal camouflage, its performance is not excellent due to its simple structure. The quantitative growth of reward during the optimization process is shown in the Fig. S5a (left), demonstrating high efficiency of the design. When the number of materials and layers increases, the performance of the optimal structure emitters improves. The performance of the six-layer and eight-layer structures even exceeds that of the five-layer structure shown in the main text. However, the promotion of structure complexity also leads to the decrease of optimization efficiency, and more structures need to be explored to reach the optimal design, as shown in Fig. S5 (right). In addition, it is worth noting that the eight-layer structure is similar to the six-layer structure in terms of materials and layer thicknesses, with the first three layers having the same materials and layer thicknesses, but the addition of ZnSe in the fourth layer leads to the differences in the subsequent structure. Since ZnSe is an infrared transparent material in the atmosphere window (AW), its presence slightly reduces the emissivity outside the AW, resulting in a slight decrease in performance compared to the six-layer structure. DQN also successfully identified this feature, making the thickness of ZnSe the minimum thickness of 200 nm that we limit, which also means increasing the number of materials or layers may not be beneficial to the design goal.

**Fig. S5 | The design results of the multilayer structure with three or four materials as a basis, including the optimal structure, corresponding emissivity spectrum and reward quantitative growth curve**. **a** Three-layer structure consisting of three materials. **b** Four-layer structure consisting of four materials. **c** Six-layer structure consisting of three stacked materials. **d** Eight-layer structure composed of four stacked materials.

**The design of the multilayer structure with different layer thickness range requirements.**

Fig. S6a shows the schematic diagram of the multilayer structure, which is a five-layer structure stacked by metal Ag and transparent dielectric. It is used to regulate the transmission and reflection properties of the solar band as a functional layer for transparent radiative cooling7. In order to guarantee the transmission in the visible band, the thickness of the Ag layer should be less than 40 nm, while the thickness of dielectric layer can be wider. The ideal transmissivity spectrum is shown in Fig. S6b. In the visible band, it has a transmissivity of 1, while in other bands including ultraviolet and near-infrared, it has a reflectance of 1. Fig. S6c shows the transmissivity spectrum of the optimal structure obtained by DQN, and it can be seen that it exhibits excellent performance, even exceeding the periodic structure proposed by Jin et al7. The optimization efficiency is also high, and only 2% of the total calculated structure needs to be explored to obtain the optimal structure, as shown in Fig. S6d.

**Fig. S6 | The design result of the multilayer structure with different layer thickness range requirements**. **a** The schematic diagram of the multilayer. **b** The ideal transmissivity spectrum. **c** The transmissivity spectrum of the optimal structure (inset). **d** Maximum of reward *R* as a function of the percentage of calculated structures.

**The design of the two-dimensional (2D) structure emitter for thermophotovoltaic**

Thermophotovoltaic (TPV) technology can output electric energy with high efficiency and power by using an emitter to convert various energy into thermal radiation matching the band gap of the TPV cell, as shown in Fig. S7a. The key to this implementation is to regulate the emissivity of the emitter to achieve high emission above the cell’s bandgap while suppressing it below bandgap. Here, we adopt the proposed framework to design a 2D periodic array structure emitter comprising a top square array, an intermediate layer and the tungsten (W) substrate, as illustrated in Fig. S7b. The materials used in the array and intermediate layer are selected from SiO2, W, Si and TiO2. Other design parameters include the thickness of the array (*t*1) and the intermediate layer (*t*2) (20-200 nm), as well as the fill rate (*γ*) of the array. The period of the whole structure is set to 600 nm. The reward *R* is set as the product of the energy conversion efficiency and the average in-band emissivity to improve both energy conversion efficiency and output power density. The specific calculations of both can be referred to our previous work8. The design results are as follows.

$R=\eta\times\varepsilon_{\text{in-band}}$ (7)

The optimal structural parameters are shown in Fig. S7b (bottom), where the materials of both the top array and the intermediate layer are chosen to be Si. The emissivity spectrum of the optimal structure is shown in Fig. S7c with a reward value of 0.5385, where the energy conversion efficiency is 0.5687 and the in-band emissivity is 0.9469. The reward as the function of the percentage of the calculated structure is shown in Fig. S7d, which demonstrates high optimization efficiency of DQN.

**Fig. S7 | Thermophotovoltaic system and the optimization results of 2D array structure emitter by DQN**. **a** Schematic of a TPV system. **b** The schematic diagram of the 2D periodic array emitter. **c** The emissivity spectrum of the optimal structure. **d** Maximum of reward *R* as a function of the percentage of calculated structures.

**References**

1. Palik, E. D. Handbook of Optical Constants of Solids. (New York: Academic Press, 1997).
2. Siefke, T. *et al*. Materials pushing the application limits of wire grid polarizers further into the deep ultraviolet spectral range. Advanced Optical Materials 4, 1780-1786 (2016).
3. Querry, M. R. Optical Constants of Minerals and Other Materials from the Millimeter to the Ultraviolet. https://apps.dtic.mil/sti/citations/ADA192210 (1987).
4. Zaman, M. A. Photonic radiative cooler optimization using Taguchi's method. *International Journal of Thermal Sciences* **144**, 21-26 (2019).
5. Motoyama, Y. *et al*. Bayesian optimization package: PHYSBO. *Computer Physic Communications* **278**, 108405 (2022).
6. Xi, W., *et al*. Colored radiative cooling: how to balance color display and radiative cooling performance. *International Journal of Thermal Sciences* **170**, 107172 (2021).
7. Jin, Y., Jeong, Y. & Yu, K. Infrared-Reflective Transparent Hyperbolic Metamaterials for Use in Radiative Cooling Windows. *Advanced Functional Materials* **33**, 2207940 (2023).
8. Hu, R. *et al*. Machine learning-optimized Tamm emitter for high-performance thermophotovoltaic system with detailed balance analysis. *Nano Energy* **72**, 104687 (2020).
